# The β-sliding clamp directs the localization of HdaA to the replisome in *Caulobacter crescentus*

**DOI:** 10.1099/mic.0.068577-0

**Published:** 2013-11

**Authors:** Carmen Fernandez-Fernandez, Karin Grosse, Victor Sourjik, Justine Collier

**Affiliations:** 1 Department of Fundamental Microbiology, Faculty of Biology and Medicine, University of Lausanne, Quartier UNIL/Sorge, Lausanne, CH 1015, Switzerland; 2 Zentrum für Molekulare Biologie der Universität Heidelberg, DKFZ-ZMBH Alliance, Im Neuenheimer Feld 282, 69120 Heidelberg, Germany

## Abstract

The initiation of chromosome replication is tightly regulated in bacteria to ensure that it takes place only once per cell cycle. In many proteobacteria, this process requires the ATP-bound form of the DnaA protein. The regulatory inactivation of DnaA (RIDA) facilitates the conversion of DnaA-ATP into replication-inactive DnaA-ADP, thereby preventing overinitiation. Homologues of the HdaA protein, together with the β-clamp of the DNA polymerase (DnaN), are required for this process. Here, we used fluorescence resonance energy transfer experiments to demonstrate that HdaA interacts with DnaN in live *Caulobacter crescentus* cells. We show that a QFKLPL motif in the N-terminal region of HdaA is required for this interaction and that this motif is also needed to recruit HdaA to the subcellular location occupied by the replisome during DNA replication. An HdaA mutant protein that cannot colocalize or interact with DnaN can also not support the essential function of HdaA. These results suggest that the recruitment of HdaA to the replisome is needed during RIDA in *C. crescentus*, probably as a means to sense whether chromosome replication has initiated before DnaA becomes inactivated. In addition, we show that a conserved R145 residue located in the AAA+ domain of HdaA is also needed for the function of HdaA, although it does not affect the interaction of HdaA with DnaN *in vivo*. The AAA+ domain of HdaA may therefore be required during RIDA after the initial recruitment of HdaA to the replisome by DnaN.

## Introduction

Proper regulation of the initiation of chromosome replication is crucial to ensure that the genome is replicated only once per cell cycle, so that cells maintain a constant number of chromosomes. In most bacterial species, each chromosome has a single origin of replication and its replication requires the initiator protein DnaA (Messer, 2002; Mott & Berger, 2007). DnaA also acts as an important transcription factor (Scholefield *et al*., 2011). Thus, it is important to understand how this protein is regulated during the bacterial cell cycle.

In the gammaproteobacterium *Escherichia coli*, DnaA or the origin of replication (*oriC*) is the target of three main systems regulating the initiation of chromosomal replication (Katayama *et al.*, 2010; Leonard & Grimwade, 2011): the titration and inactivation of DnaA molecules by the chromosomal *datA* locus (Kasho & Katayama, 2013; Kitagawa *et al.*, 1998), the sequestration of *oriC* by the SeqA protein preventing access of DnaA to *oriC* (Brendler *et al.*, 1995; Lu *et al.*, 1994; Nievera *et al.*, 2006; Slater *et al.*, 1995) and the regulatory inactivation of DnaA (RIDA) (Katayama *et al.*, 1998; Kato & Katayama, 2001). RIDA is the most important system preventing hyperinitiation and it seems to be the only of the three systems that is essential for cell growth (Camara *et al.*, 2005; Fujimitsu *et al.*, 2008; Kato & Katayama, 2001). In *E. coli*, it stimulates the intrinsic ATPase activity of the AAA+ (ATPase associated with diverse cellular activities) domain of DnaA, resulting in the conversion of ATP-DnaA into initiation-incompetent ADP-DnaA (Katayama & Crooke, 1995; Katayama *et al.*, 1998; Kato & Katayama, 2001; Su’etsugu *et al.*, 2005). In addition, the nucleotide binding to DnaA can also modify the affinity of DnaA for specific DnaA boxes located within promoter regions, thereby influencing the transcription of several DnaA-regulated genes in *E. coli* (Gon *et al.*, 2006; Olliver *et al.*, 2010; Scholefield *et al*., 2011; Speck *et al.*, 1999).

RIDA requires the activity of two proteins in *E. coli*: Hda and the DNA-loaded β-sliding clamp of the DNA polymerase (DnaN) (Katayama *et al.*, 1998; Kato & Katayama, 2001; Su’etsugu *et al.*, 2004). The Hda (homologous to DnaA) protein resembles the AAA+ domain of DnaA and is essential for the viability of *E. coli* (Kato & Katayama, 2001; Riber *et al.*, 2006). Conditional *hda* mutants accumulate too much ATP-bound DnaA and overinitiate DNA replication under restrictive conditions (Fujimitsu *et al.*, 2008; Kato & Katayama, 2001). When bound to ADP, Hda is stabilized in a monomeric state, which is supposedly the active form of the protein that can bind to DnaA (Su’etsugu *et al.*, 2008). Using a reconstituted *in vitro* RIDA system, it was also shown that the R153 residue of Hda, a putative arginine finger located in the box VII motif of the Hda AAA+ domain, is essential for hydrolysis of the ATP bound to DnaA because it promotes the interaction of Hda with DnaA (Nakamura & Katayama, 2010; Su’etsugu *et al.*, 2005, 2008). Additional experiments confirmed that Hda variants mutated in the R153 finger or in their N-terminal region cannot complement a conditional *hda* mutation, suggesting that these motifs are essential for RIDA in *E. coli* (Nakamura & Katayama, 2010). The N-terminal region of Hda contains a QLSLPL hexapeptide, which was shown to be required for the interaction between Hda and the β-sliding clamp of the DNA polymerase *in vitro* (Kurz *et al.*, 2004). Further experiments demonstrated that this interaction is a prerequisite for hydrolysis of the ATP bound to DnaA in a reconstituted *in vitro* RIDA system (Su’etsugu *et al.*, 2005). The β-sliding clamp tethers DNA polymerases onto the DNA during DNA replication. In addition, it binds to several other proteins involved in trans-lesion DNA synthesis and in DNA mismatch repair, acting as a toolbelt for the replisome (López de Saro & O’Donnell, 2001; O’Donnell, 2006). To stimulate the activity of Hda and hydrolysis of the ATP bound to DnaA *in vitro*, the β-sliding clamp must be loaded onto the DNA (Su’etsugu *et al.*, 2004), suggesting that RIDA is not active before the onset of DNA replication in *E. coli* cells.

**Fig. 1.**
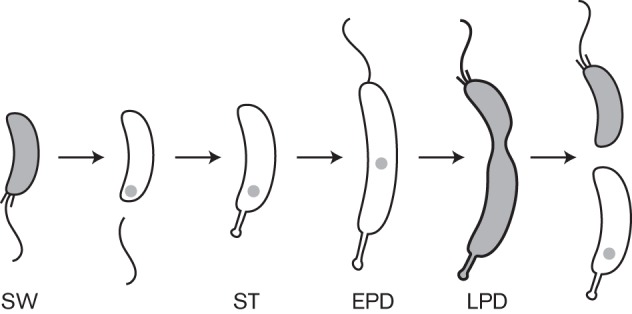
The subcellular localization of HdaA during the *C. crescentus* cell cycle. Schematic showing the *C. crescentus* cell cycle. The shading indicates the distribution of replisome components and of HdaA in cells. SW, swarmer cell; ST, stalked cell; EPD, early predivisional cell; LPD, late predivisional cell.

Studies on the regulation of the initiation of chromosomal replication in *E. coli* are sometimes complicated, because fast-growing *E. coli* cells usually contain more than two replication forks. In contrast, the alphaproteobacterium *Caulobacter crescentus* is a very convenient model to study the control of replication initiation because it initiates the replication of its chromosome only once during its cell cycle (Collier, 2012; Marczynski, 1999). *C. crescentus* divides asymmetrically, giving rise to two different cell types: a motile swarmer cell and a sessile stalked cell (Fig. 1) (Curtis & Brun, 2010). The stalked progeny immediately initiates the replication of its chromosome, while the swarmer cell cannot start the replication of its chromosome before it starts differentiating into a stalked cell (Marczynski, 1999). Using fluorescently tagged replisome components, it was shown that they are diffuse in the cytoplasm of non-replicating cells, while they form a tight fluorescent focus in replicating cells (Collier & Shapiro, 2009; Jensen *et al.*, 2001). This focus assembles on the chromosomal origin (*Cori*) at the future stalked pole of the cell at the onset of chromosome replication and then gradually moves towards mid-cell until the end of the replication process in predivisional cells (Fig. 1). Initiation of the replication of the *C. crescentus* chromosome is regulated not only by DnaA, but also by another *Cori*-binding protein, named CtrA (Collier, 2012; Domian *et al.*, 1997; Gorbatyuk & Marczynski, 2001; Quon *et al.*, 1998). In swarmer cells, CtrA binds to *Cori* and thereby inhibits the initiation of DNA replication. CtrA is nevertheless not involved in restricting origin firing to only once per cell cycle, as cells deficient in CtrA activity or lacking the five CtrA binding sites in *Cori* are not overinitiating chromosome replication (Bastedo & Marczynski, 2009; Jonas *et al.*, 2011). This control is instead dependent on the HdaA protein, which is homologous to the *E. coli* Hda protein (Collier & Shapiro, 2009). The DnaA initiator is probably inactivated by a RIDA-like mechanism in *C. crescentus*, because HdaA-depleted cells, or cells expressing a mutant DnaA protein with a disrupted ATPase domain, overinitiate chromosome replication (Collier & Shapiro, 2009; Fernandez-Fernandez *et al.*, 2011; Jonas *et al.*, 2011). As the nucleotide state of DnaA does not seem to affect the affinity of DnaA for *Cori in vitro* (Taylor *et al.*, 2011), additional factors may promote the binding of ATP-DnaA to *Cori in vivo*, or the nucleotide bound to DnaA may influence the activity of DnaA at a later step during the initiation process (Collier, 2012). In addition, the nucleotide state of DnaA may also influence the expression of several DnaA-activated genes in *C. crescentus* (Fernandez-Fernandez *et al.*, 2011).

How the activity of DnaA is spatially and temporally regulated by the RIDA process remains only partially understood in bacteria. We previously showed that fluorescently tagged HdaA colocalizes with the replisome throughout S phase in *C. crescentus* (Fig. 1) (Collier & Shapiro, 2009). It has nevertheless remained unclear (i) whether the *C. crescentus* β-sliding clamp interacts with HdaA *in vivo*, (ii) whether such an interaction is needed for the stable recruitment of HdaA at the replisome or (iii) whether the colocalization of HdaA with the replisome is necessary for RIDA in *C. crescentus*. Here we use live *C. crescentus* cells to provide answers to these questions. We then discuss the possible implications of these findings on the regulation of DNA replication and gene expression in *C. crescentus*.

## Methods

### 

#### Bacterial strains and growth conditions.

The strains used in this study are listed in Table 1. Growth conditions used in this study are described in the supplementary methods available in *Microbiology* Online.

**Table 1.** Plasmids and strains used in this study

Plasmid/strain	Description/genotype	Reference/source
**Plasmid**		
pNPTS138	Suicide vector containing the *sacB* gene; used for double homologous recombination	D. Alley (Stanford University, CA, USA)
pRXMCS-5	Low-copy number plasmid for expression of genes from *xylX* promoter	Thanbichler *et al.* (2007)
pCFPC-1	Plasmid for integrating C-terminal CFP fusions at the site of interest	Thanbichler *et al.* (2007)
pXYFPN-4	Plasmid for integrating N-terminal YFP fusions under control of the *xylX* promoter	Thanbichler *et al.* (2007)
pXYFPC-1	Plasmid for integrating C-terminal YFP fusions under control of the *xylX* promoter	Thanbichler *et al.* (2007)
pXYFPC-4	Plasmid for integrating C-terminal YFP fusions under control of the *xylX* promoter	Thanbichler *et al.* (2007)
pXCFPC-4	Plasmid for integrating C-terminal CFP fusions under control of the *xylX* promoter	Thanbichler *et al.* (2007)
pXGFP4C1	Plasmid for integrating N-terminal GFP fusions under control of the *xylX* promoter	D. Alley
pNPTS138-Δ*hdaA*	The regions upstream and downstream of *hdaA* were cloned into pNPTS138	Collier & Shapiro (2009)
pRX-HdaA	*hdaA* cloned into pRXMCS-5	This work
pRX-HdaAΔN	*hdaA*Δ*N* cloned into pRXMCS-5	This work
pRX-HdaAR145A	*hdaAR145A* cloned into pRXMCS-5	This work
pCFPC-dnaN′	The last 548 bp of *dnaN* cloned into pCFPC-1	This work
pXYFPN-HdaA	*hdaA* cloned into pXYFPN-4	This work
pXYFPN-HdaAΔN	*hdaA*Δ*N* cloned into pXYFPN-4	This work
pXYFPN-HdaAR145A	*hdaAR145* cloned into pXYFPN-4	This work
pXYFPN-CFP	*ecfp* cloned into pXYFPN-4	This work
pXYFPN-HdaA′_1–10_	The first 30 bp of *hdaA* cloned into pXYFPN-4	This work
pXYFPN-HdaA′_1–35_	The first 105 bp of *hdaA* cloned into pXYFPN-4	This work
pXYFPN-HdaA′_1–50_	The first 150 bp of *hdaA* cloned into pXYFPN-4	This work
pXGFP-HdaA	*hdaA* cloned into pXGFP4C1	Collier & Shapiro (2009)
pXGFP-HdaAΔN	*hdaA*Δ*N* cloned into pXGFP4C1	This work
pXGFP-HdaAR145A	*hdaAR145A* cloned into pXGFP4C1	This work
pLW176	CMS19	West *et al.* (2002)
***E. coli* strain**		
TOP10	Δ*hsdR* *mcrA* *lacZ*ΔM15 *endA1* *recA1*	Invitrogen
LS256	HB101 F^−^ Δ(*mcrC-mrr*) *leu* *supE44* *ara14* *galK2* *lacY1* *proA2* *rpsL20* (Strep-r) *xyl-5 mtl-1 recA13* pRK2013	Ditta *et al.* (1980)
***C. crescentus* strain**		
CB15N	Synchronizable derivative of wild-type strain CB15	Evinger & Agabian (1977)
CMS19	CB15N pLW176	West *et al.* (2002)
JC325	CB15N pNPTS138-Δ*hdaA*	Collier & Shapiro (2009)
JC570	CB15N Δ*hdaA* pRX-HdaA	This work
JC577	CB15N *dnaN* : : *dnaN*-*cfp*	This work
JC578	CB15N pXYFPN-HdaA	This work
JC593	CB15N *dnaN* : : *dnaN–cfp* pXYFPN-HdaA	This work
JC654	CB15N pXYFPN-CFP	This work
JC655	CB15N pXYFPN-HdaA′_1–10_	This work
JC673	CB15N pXYFPN-HdaA′_1–35_	This work
JC674	CB15N pXYFPN-HdaA′_1–50_	This work
JC708	CB15N *dnaN* : : *dnaN–cfp* pXYFPN-HdaAΔN	This work
JC780	CB15N pXYFPC-4	This work
JC790	CB15N Δ*hdaA* pRX-HdaA pLW176	This work
JC809	CB15N pXCFPC4 pXYFPC1	This work
JC864	CB15N Δ*hdaA* pXYFPN-HdaA	This work
JC888	CB15N *dnaN* : : *dnaN–cfp* pXYFPN-HdaAR145A	This work
JC912	CB15N pXYFPN-HdaAR145A	This work
JC1094	CB15N *dnaN* : : *dnaN–cfp* pXYFPN-HdaA’1-10	This work

#### Construction of plasmids and strains.

The plasmids and oligonucleotides used in this study are listed in Tables 1 and S1, respectively. Details of the construction of plasmids and strains are given in the supplementary methods.

#### Microscopy.

Strains were grown to exponential phase in M2G medium (Ely, 1991). Cells were immobilized onto a thin layer of M2G medium with 1 % agarose. Phase-contrast microscopy (Ph3) and fluorescence microscopy images were taken with a Zeiss AxioImager M1 microscope equipped with a Plan-Apochromat 100×/1.45 Oil Ph3 objective, a Cascade 1K EMCCD camera and filter sets for cyan fluorescent protein (CFP; excitation ET 436/20; dichroic T455LP; emission ET 480/40) and yellow fluorescent protein (YFP; excitation ET 500/20; dichroic T515LP; emission ET 535/30). Each experiment was performed at least twice using independent cultures. Imaging processing was carried out using Adobe Photoshop and Metamorph 7.5 (Universal Imaging).

#### *In vivo* acceptor photobleaching FRET measurements.

Strains were cultivated to exponential phase (OD_660_ of ~0.3) in M2G medium with 0.3 % xylose for 1 h to induce the *xylX* promoter (Meisenzahl *et al.*, 1997). A total of 5 ml of the culture was harvested by centrifugation, resuspended in 50 µl medium and cells were immobilized onto 1 % agarose pads. Acceptor photobleaching fluorescence resonance energy transfer (FRET) measurements were performed on a custom-modified Zeiss Axiovert 200 microscope as previously described (Kentner & Sourjik, 2009). Briefly, excitation light from a 75 XBO lamp, attenuated by an ND60 (0.2) neutral-density filter, passed through a band-pass (BP) 436/20 filter and a 495DCSP dichroic mirror and was reflected on the specimen by a Z440/532 dual-band beamsplitter (transmission 465–500 and 550–640 nm; reflection 425–445 and 532 nm). Bleaching of YFP was accomplished by 20 s of illumination with a 532 nm diode laser (Rapp OptoElectronic), reflected by the 495DCSP dichroic mirror into the light path. Emission from the field of view, which was narrowed with a diaphragm to the area bleached by the laser, passed through a BP 485/40 filter onto an H7421-40 photon counter (Hamamatsu). For each measurement point, photons were counted over 0.5 s using a counter function of the PCI-6034E board, controlled by a custom-written LabView 7.1 program (both from National Instruments). CFP emission was recorded before and after bleaching of YFP, and FRET was calculated as the CFP signal increase divided by the total signal after bleaching.

#### Immunoblot analysis.

Proteins were resolved by SDS-PAGE and transferred to a PVDF membrane (Millipore). Immunodetection was performed with polyclonal antibodies against the protein of interest: anti-HdaA serum was diluted 1 : 2000 and anti-GFP serum (Invitrogen) was diluted 1 : 1500. Anti-rabbit antibodies conjugated to peroxidase (Sigma-Aldrich) were used as secondary antibodies and used diluted 1 : 10 000. Signals were visualized using a chemiluminescent reagent (PerkinElmer) and Kodak Bio-Max MR films. The films were scanned and processed with Photoshop (Adobe).

## Results

### The N-terminal region of HdaA is needed for the essential function of HdaA

**Fig. 2.**
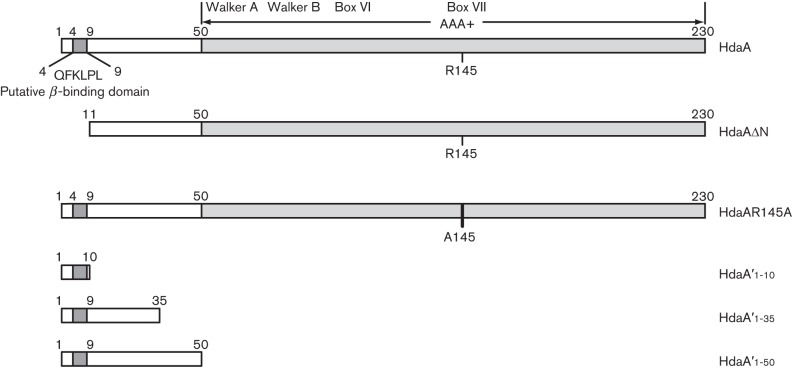
The organization of the HdaA protein. Diagram of the HdaA domain structure. Mutant and truncated HdaA proteins created for this study are also shown below the diagram of the wild-type protein.

As the *E. coli* RIDA system needs a DNA-loaded β-sliding clamp to be active *in vitro* (Katayama *et al.*, 1998; Su’etsugu *et al.*, 2004), the β-sliding clamp may be the component of the RIDA system that senses if replication is ongoing, to ensure that DnaA is inactivated only once DNA replication has started. If so, the interaction between the DNA-loaded β-sliding clamp and the Hda protein may be the signal that stimulates the activity of Hda *in vivo*. Interestingly, the N-terminal region of the *C. crescentus* HdaA protein contains a ^4^QFKLPL^9^ motif (Fig. 2) resembling the QL[SP]LPL hexapeptide that is necessary for the interaction of several proteins with the β-sliding clamp in *E. coli* (Dalrymple *et al.*, 2001; Kurz *et al.*, 2004). To determine whether this motif may be essential for RIDA in *C. crescentus*, we engineered a mutant allele of *hdaA* encoding a truncated HdaA protein that lacks its first ten amino acids (Fig. 2), expressed under the control of the xylose-inducible *xylX* promoter from a replicating plasmid (pRX-HdaAΔN). As a control, we used a similar plasmid expressing the wild-type HdaA protein (pRX-HdaA) (Collier & Shapiro, 2009). Each plasmid was introduced into strain JC325, which contained an integrated suicide vector (pNPTS138-Δ*hdaA*) that was previously constructed to create a chromosomal Δ*hdaA* mutation by double-homologous recombination and sucrose selection (Collier & Shapiro, 2009). Using the two resulting strains and strain JC325 as a control, we isolated colonies on xylose-containing medium that had excised the suicide vector. We then analysed what proportion of these colonies displayed the wild-type or the Δ*hdaA* chromosomal allele of *hdaA*. Using strain JC325 without any plasmid, we found that all colonies carried the wild-type chromosomal allele of *hdaA* (Table S2). By contrast, when we used the strain expressing the wild-type HdaA protein from pRX-HdaA, more than half of the colonies carried the chromosomal Δ*hdaA* mutation (Table S2). These observations were consistent with previous results indicating that HdaA is essential for the viability of *C. crescentus* (Collier & Shapiro, 2009). When we used the strain expressing the HdaAΔN mutant protein from pRX-HdaAΔN, all colonies carried the wild-type chromosomal allele of *hdaA* (Table S2). This result shows that the mutant HdaAΔN protein cannot compensate for the loss of HdaA, indicating that the N-terminal region of HdaA is required for the function of HdaA in *C. crescentus*.

### The N-terminal region of HdaA is needed to localize HdaA at the replisome during DNA replication

In *C. crescentus*, we previously showed that HdaA colocalizes with the β-sliding clamp. To test whether the N-terminal region of HdaA was required for this spatial association between HdaA and the replisome, we compared the subcellular localization of the wild-type HdaA protein with the mutant HdaAΔN protein in live *C. crescentus* cells.

We constructed two strains expressing a DnaN-CFP fusion protein under control of the endogenous *dnaN* promoter at the native *dnaN* locus, together with YFP–HdaA (JC593) or YFP–HdaAΔN (JC708) fusion proteins under control of the chromosomal *xylX* promoter. We then analysed these two double-labelled strains by fluorescence microscopy.

**Fig. 3.**
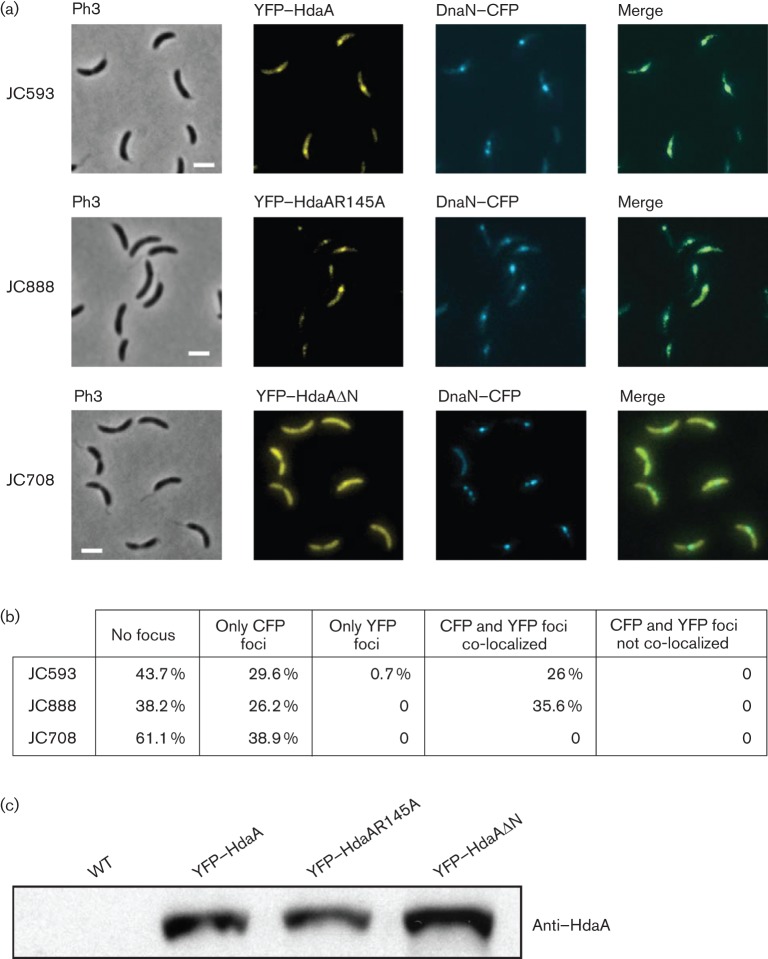
The N-terminal region of HdaA, but not its R145 finger, is necessary for the colocalization of HdaA with the β-sliding clamp of the replisome. (a) Cells from strains JC593, JC888 and JC708, expressing DnaN–CFP together with YFP–HdaA or YFP–HdaAR145A or YFP–HdaAΔN, respectively, were visualized by phase-contrast (Ph3) and fluorescence microscopy. Right panels correspond to overlays of the two fluorescence microscopy images (green colour indicates co-localization). Bars, 2 µm. (b) Statistical analysis of the results shown in (a). More than 160 cells of each strain were analysed. This table shows the percentage of cells that do not contain a fluorescent focus (no focus), that contain only DnaN–CFP foci (only CFP foci), that contain only YFP–HdaA foci (only YFP foci), that contain colocalized DnaN–CFP and YFP–HdaA foci (CFP and YFP foci colocalized) and that contain spatially dissociated DnaN–CFP and YFP–HdaA foci (CFP and YFP foci not colocalized). (c) The intracellular levels of YFP-tagged HdaA proteins in cell extracts from strains JC593, JC888 and JC708 were evaluated by immunoblot using antibodies raised against HdaA. Cells were cultivated to exponential phase in M2G medium, and 0.3 % xylose was added to the medium 1 h before sample collection for immunoblotting and fluorescence microscopy.

Using strain JC593, we observed that more than 97 % of the cells that had a detectable YFP–HdaA fluorescent focus also had a co-localized DnaN–CFP focus (Fig. 3a, b). This result confirmed previous observations that we made using a different strain expressing GFP–HdaA and DnaN–RFP fusion proteins (Collier & Shapiro, 2009). We also tested whether the YFP–HdaA protein that we engineered could replace the essential function of the native HdaA protein. To this end, we performed co-transduction experiments using ΦCR30 phages prepared from strain JC790, which carries a kanamycin-resistance marker genetically linked with a Δ*hdaA* mutation (Table 1). The Δ*hdaA* mutation was easily co-transduced with the kanamycin-resistance marker into strain JC578 expressing *yfp–hdaA* from the native *xylX* promoter. The resulting strain (JC864) grew well in rich medium, but not as well in rich medium supplemented with 0.2 % glucose (cells were very elongated), showing that *yfp–hdaA* expressed from the *xylX* promoter in the absence of glucose can complement the Δ*hdaA* mutation and, thus, that YFP–HdaA is functional *in vivo*. Nevertheless, to allow comparison with non-functional HdaA derivatives, all fluorescence microscopy experiments shown in this work were performed using merodiploid strains expressing HdaA and fluorescently tagged HdaA proteins (Table 1).

Using strain JC708, we observed that the YFP–HdaAΔN protein never formed detectable fluorescent foci in replicating cells, in contrast to the YFP–HdaA protein (Fig. 3a, b). Instead, YFP–HdaAΔN was diffuse in the cytoplasm of all cells, despite the presence of DnaN–CFP foci in 38.9 % of the cells. We confirmed that YFP–HdaA and YFP–HdaAΔN accumulated to similar intracellular levels in each strain by immunoblot analysis (Fig. 3c). Thus, the different localization patterns that we observed for YFP–HdaA and YFP–HdaAΔN were not dependent on the levels of the YFP-tagged proteins in cells. Instead, we conclude that the N-terminal region of HdaA is necessary for the colocalization of HdaA with the β-sliding clamp, and thus also for its recruitment to the replisome during ongoing DNA replication.

### HdaA interacts directly with the β-sliding clamp in live *C. crescentus* cells and this interaction requires the N-terminal region of HdaA

*In vitro* assays demonstrated that purified *E. coli* Hda proteins bind directly to DNA-loaded β-sliding clamps (Kurz *et al.*, 2004; Su’etsugu *et al.*, 2004, 2005). Similarly, yeast two-hybrid assays suggested that the *C. crescentus* HdaA protein may interact with the β-sliding clamp, in a manner that is dependent on the integrity of its N-terminal region (Jonas *et al.*, 2011). Both observations indicate that HdaA homologues may interact with the β-sliding clamp in several bacterial species, although this has, to our knowledge, not been demonstrated in live bacterial cells. If HdaA does interact with the β-sliding clamp through its N-terminal region, this interaction may explain how HdaA becomes recruited to the replisome in *C. crescentus* (Fig. 3; Collier & Shapiro, 2009).

**Fig. 4.**
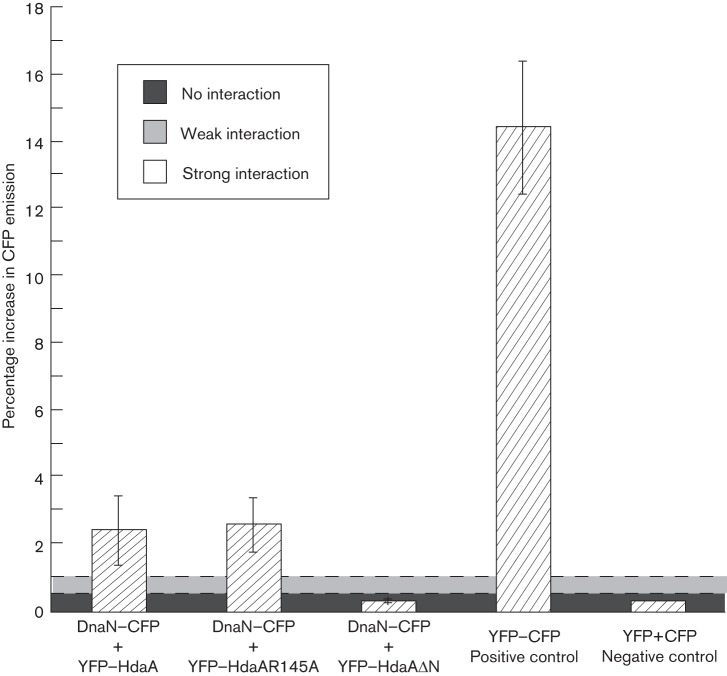
YFP–HdaA interacts with DnaN–CFP in live *C. crescentus* cells and this interaction requires the N-terminal region of HdaA but not the R145 finger of HdaA. Strains JC593, JC888 and JC708 (expressing DnaN–CFP together with YFP–HdaA or YFP–HdaAR145A or HdaAΔN, respectively), strain JC654 (expressing a YFP–CFP fusion protein) and strain JC809 (expressing YFP and CFP) were cultivated to exponential phase in M2G medium prior to the addition of 0.3 % xylose for 1 h. Live cells were then collected for acceptor photobleaching FRET analysis as previously described (Kentner & Sourjik, 2009) (also see Methods). The graph shows the mean increase in CFP emission measured after YFP photobleaching for each population of cells, corresponding to the apparent FRET efficiency. Values above 0.5 % indicate an interaction between the CFP- and the YFP-tagged proteins, while values above 1 % indicate a strong interaction (Kentner & Sourjik, 2009). As expected, the control strain JC654 gave a value above 1 %, while the control strain JC809 gave a value below 0.5 %. For each strain, at least three measurements were performed. Error bars indicate sd.

To determine if HdaA interacts with the β-sliding clamp in live *C. crescentus* cells, we used acceptor photobleaching FRET experiments. FRET is based on the distance-dependent energy transfer from an excited donor to an acceptor fluorophore and it allows the detection of intracellular interactions between fluorescently labelled proteins (Miyawaki & Tsien, 2000; Sourjik & Berg, 2002; Wouters & Bastiaens, 2001). In acceptor photobleaching FRET experiments, selective bleaching of the acceptor (YFP) causes a detectable unquenching of the donor (CFP) emission if the two fluorophores are in the immediate proximity. This sensitive method can detect even transient interactions in live bacterial cells (Kentner & Sourjik, 2009). To test whether HdaA–YFP interacts with DnaN–CFP, we used the same strain (JC593) as used for the co-localization studies (Fig. 3). Our measurements demonstrate that YFP–HdaA and DnaN–CFP interact strongly, and probably directly, in *C. crescentus* (Fig. 4). Using strain JC708, expressing YFP–HdaAΔN instead of YFP–HdaA, we detected only a weak signal that was comparable to that of the negative control strain JC809, expressing YFP and CFP from the chromosomal *xylX* promoter (Fig. 4). This result demonstrates that the N-terminal region of HdaA is required for the DnaN–HdaA interaction. As the co-localization of HdaA with the replisome was also lost when the N-terminal region of HdaA was removed (Fig. 3), it is likely that the recruitment of HdaA to the replisome is dependent on the interaction between HdaA and DnaN, and that this interaction is essential for RIDA *in vivo* (Table S2).

### The N-terminal region of HdaA is not sufficient to localize YFP to the β-sliding clamp *in vivo*

A 10 aa peptide containing the putative β-binding motif of the *E. coli* Hda protein was previously shown to compete with purified Hda for binding to the β-sliding clamp (Kurz *et al.*, 2004). This observation suggests that this motif is sufficient to interact with the β-sliding clamp, at least *in vitro*. We therefore wished to determine if different N-terminal fragments of HdaA containing the putative β-binding motif were sufficient to recruit YFP to the replisome in live *C. crescentus* cells.

**Fig. 5.**
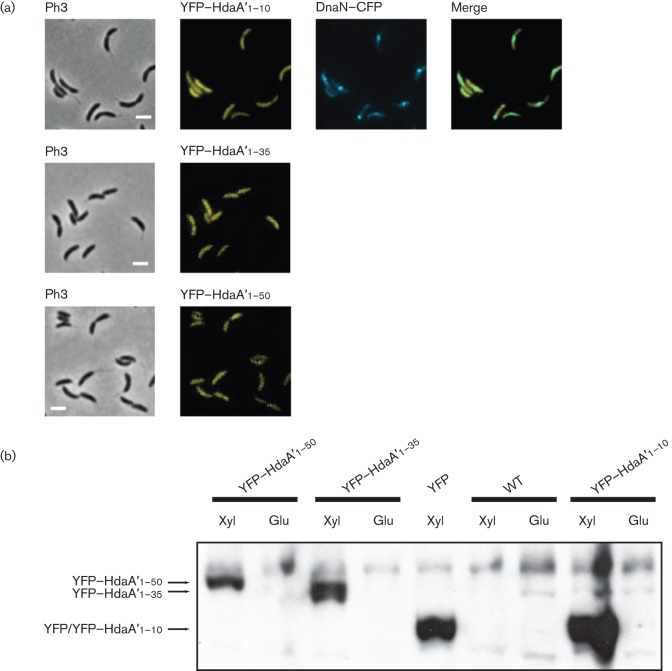
The N-terminal region of HdaA is not sufficient for co-localization with the replisome. (a) Cells of strains JC1094 (expressing DnaN–CFP and YFP–HdaA′_1–10_), JC673 (expressing YFP–HdaA′_1–35_) and JC674 (expressing YFP–HdaA′_1–50_) were cultivated to exponential phase in M2G medium, and 0.3 % xylose was added to the medium 1 h before sample collection. Cells were visualized by phase-contrast (Ph3) and fluorescence microscopy. For strain JC1094, the right panel shows an overlay of the two fluorescence microscopy images. Bars, 2 µm. (b) The intracellular levels of YFP-tagged HdaA′ proteins in cell extracts from strains JC1094, JC673, JC674, JC780 (expressing YFP) and CB15N (wild-type, WT) were analysed by immunoblot using antibodies raised against GFP (also detecting YFP). Cells were cultivated to exponential phase in M2G medium and 0.3 % xylose (Xyl) or 0.2 % glucose (Glu) was added to media 6 h before sample collection.

We constructed three strains expressing the YFP protein fused to N-terminal HdaA fragments of different lengths (Fig. 2) under control of the chromosomal *xylX* promoter. The shortest construct, expressing YFP fused to the first 10 aa of HdaA (YFP–HdaA′_1–10_), was introduced into a strain that was also expressing DnaN–CFP, resulting in strain JC1094. Using fluorescence microscopy, we observed that YFP–HdaA′_1–10_ never formed fluorescent foci, even in replicating cells that contained a DnaN–CFP focus (Fig. 5a). Instead, the fluorescence signal from YFP–HdaA′_1–10_ was diffusely distributed in the cytoplasm of cells (Fig. 5a), like that of the YFP protein alone (data not shown). This result shows that the first 10 aa of the HdaA protein are necessary (Fig. 3a) but not sufficient (Fig. 5a) for co-localization with the β-sliding clamp. The other two constructs, expressing YFP fused to the first 35 or 50 aa of HdaA (YFP–HdaA′_1–35_ and YFP–HdaA′_1–50_, respectively), were introduced into a wild-type strain, resulting in strains JC673 and JC674, respectively. We observed that the fluorescent signals from YFP-HdaA′_1–35_ and YFP-HdaA′_1–50_ were also diffusely distributed in the cell cytoplasm. To verify that these HdaA N-terminal fragments were not cleaved off from YFP *in vivo*, we performed immunoblot experiments using protein extracts from the two strains (Fig. 5b). We also estimated the relative abundancy of YFP–HdaA′_1–10_ compared with HdaA in cells from the merodiploid strain JC1094 by immunoblot analysis and found that YFP–HdaA′_1–10_ accumulates at much higher levels than HdaA (Fig. S1). Thus, it is very unlikely that HdaA simply outcompetes YFP–HdaA′_1–10_ when binding to DnaN. We conclude that the 50 N-terminal amino acids of HdaA are not sufficient for the recruitment of YFP to the replisome (Fig. 5a), although the ten N-terminal amino acids of HdaA are required for the recruitment of HdaA to the replisome (Fig. 3).

### The R145 finger of HdaA is needed for the essential function of the protein, but it is not involved in the interaction with the β-sliding clamp or in focus formation

As we showed that the presence of the ^4^QFKLPL^9^ motif is not sufficient to localize YFP to the β-sliding clamp, there may be other motifs in HdaA that promote or stabilize the recruitment of HdaA to the replisome. We therefore looked for other domains of HdaA that may be involved in protein/protein interactions. The conserved R145 finger in box VII of the AAA+ domain of HdaA was an interesting candidate. This residue corresponds to the R153 residue of *E. coli* Hda, which is involved in interactions with DnaA during RIDA (Nakamura & Katayama, 2010), consistent with the frequent roles of AAA+ domains in intra- or intermolecular interactions *in vivo* (Neuwald *et al.*, 1999; Ogura *et al.*, 2004). To investigate the potential role of the conserved R145 finger of HdaA in *C. crescentus*, we tested whether an HdaAR145A mutant protein was functional *in vivo* and whether it was interacting and co-localizing with the β-sliding clamp.

We first created a plasmid expressing the mutant HdaAR145A protein (Fig. 2) from the *xylX* promoter (pRX-HdaAR145A) and introduced this plasmid into strain JC325, as previously done with the pRX-HdaA and pRX-HdaAΔN plasmids. Upon re-excision of the suicide vector, we exclusively recovered the wild-type allele of the *hdaA* gene (Table S2), demonstrating that the expression of HdaAR145A cannot compensate for the loss of *hdaA*. We concluded that the R145 finger of HdaA is needed for the function of HdaA in *C. crescentus*.

We next created a strain (JC888) that expressed YFP–HdaAR145A from the chromosomal *xylX* promoter and DnaN–CFP from the native *dnaN* promoter and analysed these cells by fluorescence microscopy and by FRET. We found that YFP–HdaAR145A was interacting with DnaN–CFP as efficiently as YFP–HdaA (Fig. 4), demonstrating that the R145 finger of HdaA is not required for a stable interaction between HdaA and the β-sliding clamp *in vivo*. Interestingly, the YFP–HdaAR145A protein also co-localized with DnaN–CFP (Fig. 3), suggesting that the R145 finger of HdaA is also not required for interaction with a replisome component other than the β-sliding clamp, which could have stabilized the spatial association between HdaA and the replisome *in vivo*. We conclude that the R145 finger of HdaA is not necessary for the recruitment of HdaA to the replisome, although it is needed for the essential function of the protein in *C. crescentus*.

### The R145 finger reduces the accumulation of HdaA molecules at the replisome

**Fig. 6.**
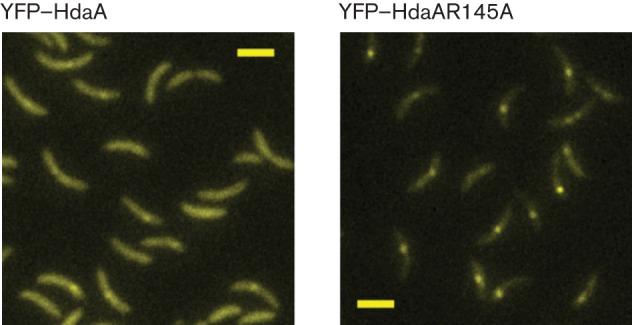
Mutant YFP–HdaAR145A proteins form brighter fluorescent foci than YFP–HdaA in replicating predivisional cells. Swarmer cells from cultures of strains JC578 (expressing YFP–HdaA) and JC912 (expressing YFP–HdaAR145A) were isolated by centrifugation in a Percoll density gradient (Evinger & Agabian, 1977), resuspended in M2G medium containing 0.15 % xylose and allowed to progress synchronously through their cell cycle. Samples of each culture were collected after 120 min. The resulting predivisional cells were then visualized by fluorescence microscopy (same greyscale). Representative images acquired and visualized using identical settings are shown. Bars, 2 µm.

When we compared unsynchronized *C. crescentus* cultures of cells expressing YFP–HdaA (JC578) or YFP–HdaAR145A (JC912), we noted that YFP–HdaAR145A foci appeared brighter than YFP–HdaA foci (Fig. 3a). To better characterize this difference, we isolated swarmer cells of each strain and grew them to the predivisional stage in minimal medium for 120 min: DNA replication then took place in most cells, facilitating the analysis of replisome-associated YFP–HdaA and YFP–HdaAR145A foci. Fluorescence microscopy images acquired and visualized using identical settings confirmed that YFP–HdaAR145A foci appeared much brighter than YFP–HdaA in replicating predivisional cells (Fig. 6). A quantitative analysis of the fluorescence intensity of foci from hundreds of cells demonstrated that the difference was significant (Fig. S2) and independent of the slight differences in intracellular levels of YFP–HdaA and YFP–HdaAR145A (data not shown). We conclude that more YFP-HdaAR145A than YFP-HdaA molecules were co-localized with the replisome in live cells, suggesting that the R145 finger of HdaA may prevent the oligomerization of HdaA molecules at the replisome.

## Discussion

### The necessary recruitment of HdaA to the replisome

Our previous studies showed that the HdaA protein formed a complex with the replisome throughout the S phase of the *C. crescentus* cell cycle (Fig. 1) (Collier & Shapiro, 2009). In the present report, we provide evidence showing that HdaA is recruited to the replisome through a direct interaction with the β-clamp of the DNA polymerase. We used functional fluorescently tagged proteins to demonstrate that this interaction takes place in live *C. crescentus* cells. Our results show that this interaction and the localization of HdaA at the replisome are both dependent on the presence of a short motif at the N-terminal region of HdaA, but not on the conserved R145 finger in the AAA+ domain of HdaA (Figs 3 and 4). Interestingly, neither this short N-terminal motif nor longer N-terminal motifs of HdaA were sufficient to recruit the YFP protein to the replisome (Fig. 5). This last observation implies that other motifs in the HdaA protein, or a specific spatial conformation of the complete protein, may be required to stabilize HdaA at the replisome *in vivo*. Alternatively, YFP may have hidden the N-terminal motif of truncated HdaA proteins, preventing them from interacting with DnaN. When we tested the functionality of a mutant HdaA protein that could not localize to the replisome due to the truncation of its ten N-terminal amino acids, we found that it could not compensate for the loss of the wild-type HdaA protein (Table S2). This result suggests that HdaA is not functional when it is not localized at the replisome, indicating that the interaction with the DNA-loaded β-clamp may promote the essential activity of HdaA, as previously proposed for the Hda protein in *E. coli* (Katayama *et al.*, 1998; Kato & Katayama, 2001; Kurokawa *et al.*, 1999). This may ensure that HdaA does not promote the conversion of replication-efficient DnaA-ATP into replication-inefficient DnaA-ADP before the replication of the chromosome was initiated. Consistent with this hypothesis, we propose that the activity of HdaA is tightly regulated by the β-clamp of the DNA polymerase, so that it gets turned on only after the onset of the replication process, to prevent overinitiation without preventing the first initiation event during the *C. crescentus* cell cycle. Interestingly, the nucleotide bound to DnaA may also influence the activity of DnaA when acting as a transcription factor regulating the expression of several essential genes in *C. crescentus* (Fernandez-Fernandez *et al.*, 2011). Therefore, the temporal regulation of the activity of HdaA by the β-clamp may also play a role in coordinating the expression of these essential genes with the initiation of DNA replication.

### The AAA+ domain of HdaA

Our study demonstrates that the conserved R145 finger of the AAA+ domain of HdaA is also needed for the essential activity of HdaA at the replisome (Table S2). We showed that this residue was, however, not required for the formation of the β-clamp–HdaA complex *in vivo* (Figs 3 and 4). These two observations suggest that the AAA+ domain of HdaA is essential for the function of HdaA after its initial recruitment onto the replisome. Although the R finger of HdaA could be involved in another type of protein interaction, our data suggest that the colocalization of HdaA and DnaN is not dependent on it. Interestingly, we found that mutant YFP–HdaAR145A molecules formed more fluorescent foci than wild-type YFP–HdaA molecules in live replicating cells (Figs 6 and S2). This observation suggests that the AAA+ domain of HdaA affects the accumulation of HdaA at the replisome, despite the fact that its integrity is not required for the colocalization of HdaA with the replisome. AAA+ domains are often involved in protein–protein interactions and it was previously shown that purified *E. coli* Hda that was mutated in the corresponding R153 finger formed homo-multimers *in vitro* (Su’etsugu *et al.*, 2005). Consistent with this and with our fluorescence microscopy observations (Figs 6 and S2), we propose that the AAA+ domain of HdaA may also downregulate the oligomerization of HdaA. Besides, the R153 finger of *E. coli* Hda seemed to be required for the interaction between Hda and DnaA, and for the subsequent activation of the ATPase activity of DnaA in reconstituted RIDA systems (Nakamura & Katayama, 2010). In *C. crescentus*, the R145 finger of HdaA could similarly be needed for interaction with DnaA, which may explain why the HdaAR145A protein could not compensate for the loss of HdaA (Table S2). Further investigations will be needed to better understand the role of the AAA+ domains of Hda and HdaA in the RIDA process in *E. coli* and *C. crescentus*. This topic is of general interest as many other prokaryotic and eukaryotic AAA+ proteins play important roles during the initiation of DNA replication (Davey *et al.*, 2002; Duderstadt & Berger, 2008) and little is known about the function of many AAA+ domains in these multiprotein complexes.

### The essential function of HdaA in *C. crescentus*

A recent study of the *E. coli* Hda protein suggested that the essential function of Hda may be not to inactivate DnaA (Baxter & Sutton, 2012), but rather to influence the recruitment of alternative DNA polymerases to the β-clamp (Baxter & Sutton, 2012) or to regulate the interaction of the β-clamp with other replication proteins, such as TrfA (Kim *et al.*, 2003). If HdaA also has such additional functions, these may require the co-localization of HdaA with the β-clamp throughout the S-phase in *C. crescentus* (Collier & Shapiro, 2009). Of note, mutations in the N-terminal motif of Hda (Nakamura & Katayama, 2010) or HdaA (Table S2) were lethal in all cases tested, suggesting that the essential function of these proteins is always dependent on an interaction with the β-clamp. Accordingly, the essential function of HdaA could be linked with its capacity to compete with other proteins to restrict their access to the β-clamp. We nevertheless think that this is unlikely, as the mutant HdaAR145A protein, which interacts with the β-clamp (Fig. 4), cannot support the essential function of HdaA (Table S2). Instead, we propose that the essential function of HdaA requires its functional AAA+ domain, acting downstream of the recruitment of HdaA to the replisome. Mutant HdaAR145A molecules that tend to overaccumulate at the β-clamp (Figs 6 and S2) might nevertheless affect putative interactions between the β-clamp and other proteins.

Another very recent study demonstrated that there exists a second mechanism promoting hydrolysis of the ATP bound to DnaA in *E. coli* (Kasho & Katayama, 2013), which involves the *datA* chromosomal locus. To date, there is no evidence that equivalent functional *datA* loci exist on the *C. crescentus* chromosome. RIDA involving HdaA may therefore be essential in *C. crescentus*, consistent with the finding that hydrolysis of the ATP bound to DnaA is essential in this species (Fernandez-Fernandez *et al.*, 2011). Despite multiple similarities, it seems that different bacterial species possess different redundant systems to ensure the precise control of the replication of their chromosome(s), showing that it is important to study these control systems in a variety of bacteria. A regulatory system that represses the activity of initiator proteins and that is spatially associated with the replication fork seems generally present in distantly related bacteria, and even in eukaryotes, as is the case during RIDA in proteobacteria (Kim & Kipreos, 2007; Nishitani *et al.*, 2006).
